# Characteristics and Functions of the Yip1 Domain Family (YIPF), Multi-Span Transmembrane Proteins Mainly Localized to the Golgi Apparatus

**DOI:** 10.3389/fcell.2019.00130

**Published:** 2019-07-30

**Authors:** Shaheena Shaik, Himani Pandey, Satish Kumar Thirumalasetti, Nobuhiro Nakamura

**Affiliations:** ^1^Graduate School of Life Sciences, Kyoto Sangyo University, Kyoto, Japan; ^2^Faculty of Life Sciences, Kyoto Sangyo University, Kyoto, Japan; ^3^Department of Biotechnology, Vignan’s University, Guntur, India

**Keywords:** membrane traffic, interactome, Rab/Ypt proteins, ER–Golgi transport, vesicle budding, vesicle fusion

## Abstract

Yip1 domain family (YIPF) proteins are multi-span, transmembrane proteins mainly localized in the Golgi apparatus. YIPF proteins have been found in virtually all eukaryotes, suggesting that they have essential function(s). *Saccharomyces cerevisiae* contains four YIPFs: Yip1p, Yif1p, Yip4p, and Yip5p. Early analyses in *S. cerevisiae* indicated that Yip1p and Yif1p bind to each other and play a role in budding of transport vesicles and/or fusion of vesicles to target membranes. However, the molecular basis of their functions remains unclear. Analysis of YIPF proteins in mammalian cells has yielded significant clues about the function of these proteins. Human cells have nine family members that appear to have overlapping functions. These YIPF proteins are divided into two sub-families: YIPFα/Yip1p and YIPFβ/Yif1p. A YIPFα molecule forms a complex with a specific partner YIPFβ molecule. In the most broadly hypothesized scenario, a basic tetramer complex is formed from two molecules of each partner YIPF protein, and this tetramer forms a higher order oligomer. Three distinct YIPF protein complexes are formed from pairs of YIPFα and YIPFβ proteins. These are differently localized in either the early, middle, or late compartments of the Golgi apparatus and are recycled between adjacent compartments. Because a YIPF protein is predicted to have five transmembrane segments, a YIPF tetramer complex is predicted to have 20 transmembrane segments. This high number of transmembrane segments suggests that YIPF complexes function as channels, transporters, or transmembrane receptors. Here, the evidence from functional studies of YIPF proteins obtained during the last two decades is summarized and discussed.

## YIPF Proteins of *Saccharomyces cerevisiae* and Their Proposed Functions

Yip1 domain family proteins are multi-span transmembrane proteins localized mainly to the Golgi apparatus. Yip1p, a founding member of the YIPF proteins, was found to interact with Ypt1p and Ypt31p, which are homologs of mammalian Rab1 and Rab11, respectively (Yang et al., 1998). Ypt1p and Ypt31p are Ypt/Rab family small GTPases localized at the Golgi apparatus, and essential for ER to Golgi and intra-Golgi transport in *Saccharomyces cerevisiae* (Lipatova et al., 2015). Yip1p was shown to be required for ER to Golgi and intra-Golgi transport consistent with the proposed functions of Ypt1p and Ypt31p (Yang et al., 1998). Furthermore, a temperature sensitive mutant of Yip1p showed synthetic lethality with Ypt1p and Ypt31p, strongly suggesting that the interactions of Yip1p with Ypt1p and Ypt31p are essential for ER to Golgi transport. Later, Yif1p was found to interact with Yip1p (Matern et al., 2000). Yif1p formed a complex with Yip1p, also interacted with Ypt1p and Ypt31p, and was similarly shown to be essential for ER to Golgi transport. Based on these results, it was proposed that the Yip1p–Yif1p complex binds Ypt1p and Ypt31p to play an essential role(s) in ER to Golgi transport (Matern et al., 2000).

Yip1p and Yif1p both have paralogs, Yip4p and Yip5p, respectively. Yip4p and Yip5p were found to interact with Yip1p and Yif1p and also with Ypt/Rab GTPases (Calero et al., 2002). In contrast with Yip1p and Yif1p, Yip4p and Yip5p were found to be non-essential for viability (Giaever et al., 2002; Pearson and Schweizer, 2002), but the loss of Yip4p and Yip5p did produce some notable phenotypes, including abnormal vacuolar morphology and decreased endocytosis, suggesting a significant role in the membrane trafficking pathway, likely at the late Golgi compartment (Burston et al., 2009; Michaillat and Mayer, 2013). Yip5p strongly interacted with Yip4p, suggesting that these two proteins function together in a complex, similar to Yip1p and Yif1p (Calero et al., 2002). This idea is supported by our biochemical analysis of human homologs, YIPF1, YIPF2, and YIPF6 (Soonthornsit et al., 2017), and a phylogenetic analysis, which is described later. The loss of Yip1p could be compensated for by overexpression of Yif1p, but not by Yip4p, suggesting that Yip1p–Yif1p and Yip4p–Yip5p have non-overlapping function(s) (Calero et al., 2002).

**FIGURE 1:**
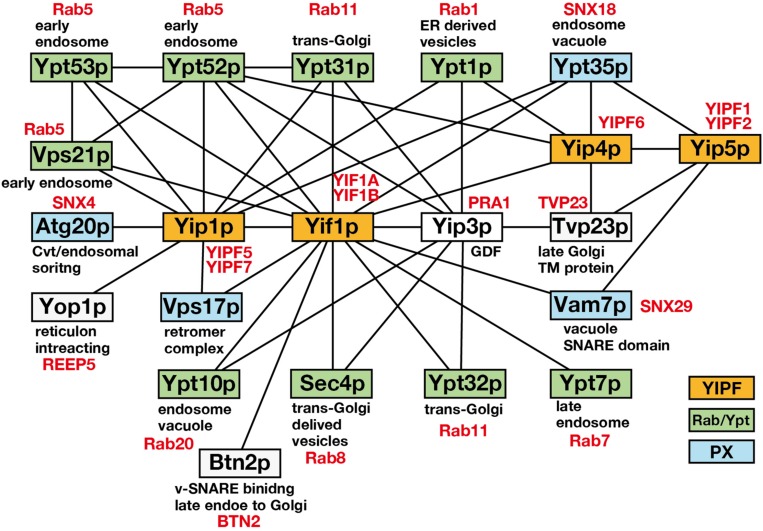
The core physical interaction network consisting of Yip1p, Yif1p, Yip4p, and Yip5p. A core physical interaction network of *S. cerevisiae* Yip1p, Yif1p, Yip4p, and Yip5p is shown. Interactions detected by more than two independent analyses were selected from SDG (Table 1) and only the gene products related to membrane trafficking are shown. Solid lines connecting boxes indicate the interaction. YIPF, Ypt/Rab, and PX domain containing proteins are colored in orange, green, and blue, respectively. Gene products are annotated for their functions or localization. Human orthologs are indicated in red according to Buvelot Frei et al. (2006), Teasdale and Collins (2012), and Lipatova et al. (2015).

It has been shown that Ypt1p functions in ER to Golgi and intra-Golgi transport at the vesicle docking/fusion step, while Ypt31p functions in *trans*-Golgi to plasma membrane transport and in endosome to *trans*-Golgi transport at the vesicle budding step (Lipatova et al., 2015). Therefore, YIPF proteins are proposed to function in vesicle budding and/or fusion at the Golgi apparatus. This is supported by the results of interactome analyses, which showed that YIPF proteins (Yip1p, Yif1p, Yip4p, Yip5p) form a core physical interaction network with selections of Ypt/Rab GTPases (Figure 1). This core network is connected with other gene products that function in membrane trafficking, including SNAREs, COPII components (Table 1; Ito et al., 2000; Uetz et al., 2000; Heidtman et al., 2003; Chen et al., 2004; Vollert and Uetz, 2004; Inadome et al., 2007; Lorente-Rodríguez et al., 2009). Furthermore, genes that function in membrane trafficking have been shown to interact genetically with *YIP1*, *YIF1*, *YIP4*, and *YIP5* (Supplementary Table S1). These include COPI and COPII coats, vesicle tethering factors (COG, TRAPP, and GARP complexes), proposed cargo receptors (p23 members), and regulators of Ypt/Rab and ARF family GTPases (ARFGAPs, GEFs for Ypt/Rab) (Supplementary Table S1; refer SDG; *Saccharomyces* Genome Database^1^).

**TABLE 1:** Physical interactors of budding yeast YIPF proteins.

**Category**	**Protein name**
Ypt	**Sec4p**, **Vps21p**, **Ypt1p**, **Ypt6p**, Ypt7p, Ypt10p, Ypt11p, **Ypt31p**, **Ypt52p**, Ypt53p
PX domain	Atg20p, Snx3p, Snx4p, Snx41p, Vam7p, Vps5p, Vps17p, Ypt35p
Ypt related	Muk1p
SNARE	Bos1p, Sed5p
COPII	**Sec13p**, **Sec23p**, **Sec24p**
COPII cargo	**Yos1p**, Yip3p
ARFGAP	**Gcs1p**
p23	Erp1p
Golgi localized	Psg1p
Membrane traffic	Btn2p, Sro77p, Tvp23p, Vps34p
ER localized	Gtt1p, Mga2p, **Yop1p**
Autophagy	Atg9p, Atg23p, **Vps30p**
Glycosylation	Ost4p
Lipid metabolism	Eeb1p
Peroxisome	Pex29p
Mitochondria	Coq5p, Pkp2p
Signal transduction	Bck1p, **Sst2p**, Tpk2p, Tpk3p
Cell cycle	Pho85
Ub/proteasome	Rsp5p
RNA binding	Bfr1, Mpt5, Nab2, Puf2, Puf3, Puf4, Slf1, Sro9
Transcription	Ccr4, Gis2
Other	Atf2, Ctf19, Hsp82, Ifa38, Itr2, Ssb2, Uip3

*Physical interactors of all YIPF proteins were combined and categorized according to functional significance. The genetic interactors for YIPF genes are indicated by bold letters. Refer SGD for the full dataset and additional information.*

Involvement of Yip1p and Yif1p in vesicle budding/fusion was evaluated by an *in vitro* vesicle budding and fusion assay by two independent groups. Ferro-Novick’s group reported that antibodies for Yip1p and Yif1p inhibited vesicle fusion to the Golgi apparatus, but neither vesicle budding from the ER nor vesicle packaging of Yip1p and Yif1p were affected (Barrowman et al., 2003). The antibody must be added at the vesicle budding step for inhibition, suggesting that the Yip1p–Yif1p complex is involved in establishing fusion competence of ER to Golgi transport vesicles at the vesicle budding step. In contrast, Barlowe’s group, using a similar *in vitro* assay, reported that antibodies against Yip1p inhibited COPII vesicle budding from the ER, but not tethering or fusion of the vesicles to Golgi membranes (Heidtman et al., 2003). *yip1-4*, a temperature sensitive mutant of *YIP1*, did not cause vesicle accumulation at a restrictive temperature, supporting their *in vitro* assay results.

Analysis of Yip1A, mammalian homolog of Yip1p, supported its function in vesicle budding (discussed later) (Tang et al., 2001). In addition, *YIP1* genetically interacts with *GOT1*, which is a tetra-spanning small membrane protein that is predicted to function in ER to Golgi transport at the vesicle budding step (Lorente-Rodríguez et al., 2009). *GOT1* was identified as a multicopy suppressor of *yip1-2*, which is a temperature sensitive mutant of *YIP1*. Got1p cycles between the ER and the Golgi apparatus, and its overexpression causes a complex extension of the ER membrane and simultaneous disruption of the Golgi apparatus, suggesting it functions at either the ER export step or vesicle budding. However, Got1p was not found to form a stable complex with Yip1p, and deletion of *GOT1* did not affect the localization of Yip1p. Therefore, the mechanism for the suppression of Yip1p function appears to be indirect, and the relationship of Got1p and Yip1p still requires further clarification.

Because Yip1p and Yif1p are efficiently packaged into COPII vesicles, they must recycle between the ER and the Golgi apparatus (Otte et al., 2001; Barrowman et al., 2003). Considering their interaction with SNAREs, COPI and COPII coats, and other associated factors that regulate the ER to Golgi transport as described above, it is reasonable to assume that Yip1p and Yif1p play roles in both vesicle budding and fusion to coordinate vesicle flow between the ER and the Golgi apparatus.

Interestingly, YIPF proteins also interacted with Atg20p, Ypt35p, Vam7p, and Vps17p, which are PX domain containing proteins (Figure 1; Vollert and Uetz, 2004). The PX domain binds phosphatidylinositol 3-phosphate and functions to recruit the proteins to endosomal membranes (Teasdale and Collins, 2012). Most of the PX domain containing proteins are classified as sorting nexins (SNXs). SNXs bind to retromer components that function in the recycling of cargo proteins from endosomes to TGN or plasma membrane (Gallon and Cullen, 2015). Therefore, it is possible that YIPF proteins function also in the endosome to TGN transport. However, the significance of the binding between YIPF proteins and SNXs has not been analyzed so far.

## Conservation of *YIP1P*, *YIF1P* Homologs in Eukaryotes

**FIGURE 2:**
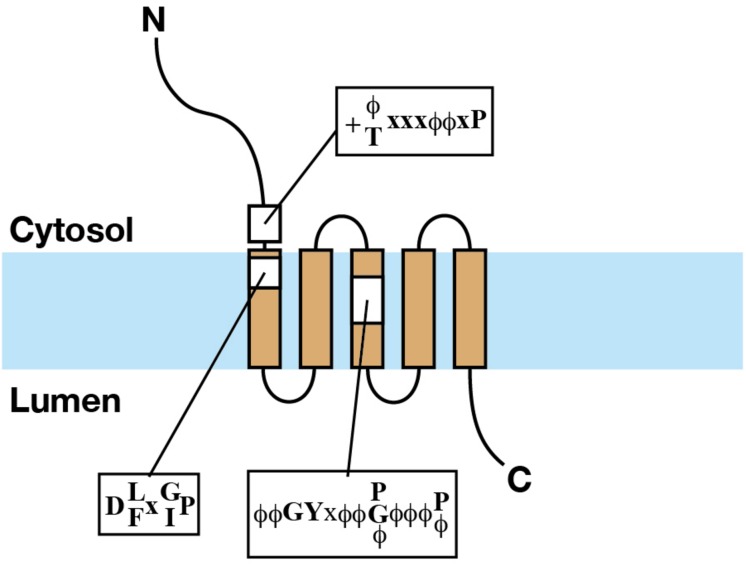
The structure of YIPF proteins. Schematic representation of the structure of a YIPF protein. The light blue band indicates the lipid bilayer. Brown squares connected by solid lines indicate transmembrane segments. The regions of the three conserved motifs are shown by yellow squares and the consensus sequences were shown (refer the text for the explanation of the motifs).

Our early BLAST search analysis identified mammalian homologs of *YIP1*, *YIF1*, *YIP4*, and *YIP5* from the protein sequence and EST databases (Shakoori et al., 2003). Multiple sequence alignment revealed that these proteins, now called the YIPF proteins, commonly have multiple hydrophobic segments with scattered hydrophilic residues on their C-terminal side (a revised protein sequence alignment result is shown in Supplementary Figure S1). Many of these proteins were predicted to have five transmembrane helices by membrane topology analyses using TMpred or SOSUI (Hofmann and Stoffel, 1993; Hirokawa et al., 1998), although some were predicted to have three transmembrane helices, likely because of a rather high number of hydrophilic residues in the hydrophobic segments. Yeast two hybrid analyses indicated that the N-termini of yeast and human YIPF proteins are exposed to the cytoplasm because N-terminal tagging of Gal4 domains showed interaction with similarly tagged cytoplasmic Ypt proteins (Yang et al., 1998; Matern et al., 2000; Calero et al., 2002; Shakoori et al., 2003). Biochemical analyses showed that all of the examined human YIPF proteins exposed their N-terminal hydrophilic regions to the cytosol and short C-terminal hydrophilic regions to the lumen of the Golgi apparatus (Figure 2; Tang et al., 2001; Shakoori et al., 2003; Yoshida et al., 2008; Tanimoto et al., 2011). From these results, it was predicted that YIPF proteins have an odd number of transmembrane segments, most probably five, with an N-terminal cytoplasmic region exposed to the cytoplasm and a short C-terminal region exposed to the lumen of the Golgi apparatus (Shakoori et al., 2003). The transmembrane region is composed of multiple hydrophobic segments and is well conserved within YIPF proteins, while the N- and the C-terminal regions are less conserved (Supplementary Figure S1). The conservation of the transmembrane region was confirmed by bioinformatics and is now annotated as “Yip1 domain” in CDD (Marchler-Bauer et al., 2017). Thus, human homolog proteins were called the “YIPF” by the HUGO gene nomenclature committee, except YIF1A and YIF1B which are homologs of *S. cerevisiae* Yif1p (Yoshida et al., 2008; Marchler-Bauer et al., 2017).

Yip1 domain family protein sequences were found in virtually all eukaryotes including protists, fungi, animals, and plants (Table 2 and Supplementary Table S2). To our surprise, proteins containing a domain similar to YIPF are even found in prokaryotes belonging to the phylum euryarchaeota (COG2881), and bacteria, including *Escherichia coli* (pfam06930: DUF1282) (Supplementary Table S3; Makarova et al., 2015; Marchler-Bauer et al., 2017). Many of these proteins are now annotated as “Yip1 family protein,” although these prokaryotic protein sequences were distantly related to eukaryotic family members (Supplementary Figures S1, S2). No function has been reported for these prokaryotic family members, and the significance of their similarity to the eukaryotic family members requires further investigation.

**TABLE 2:** Conservation of YIPF proteins in holozoan species.

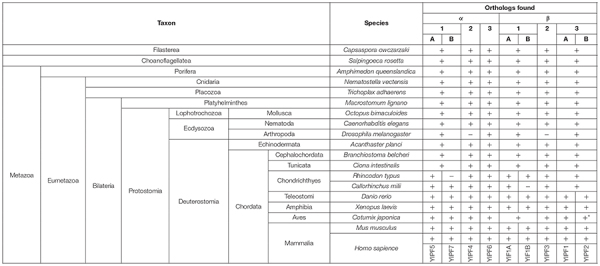

*Orthologs for each YIPF family member were BLAST searched in the NCBI database (March 2019), limiting the indicated taxa, and multiple alignment of the selected sequences was performed with mammalian family members to identify an ortholog for each family member. +, the presence of the orthologs in most of the species in the indicated taxa; an asterisk, the presence of the orthologs in only limited species in the taxon; −, no ortholog found.*

**FIGURE 3:**
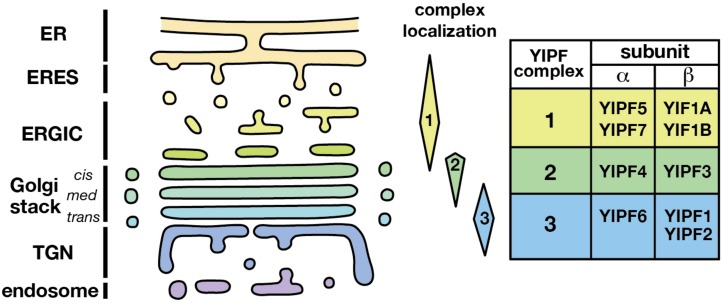
Distinct localization of YIPF complexes. The localization of three distinct YIPF complexes was shown schematically. Complexes 1, 2, and 3 are mainly localized at the early, middle, and late compartments of the Golgi apparatus, respectively. The human YIPF component proteins of each complex are shown on the right. Refer Table 3 for the synonyms of YIPF proteins.

Phylogenetic analysis after multiple sequence alignment using CLUSTALW revealed that YIPF proteins were divided into two large subfamilies represented by *S. cerevisiae* Yip1p and Yif1p, respectively (Supplementary Figure S2). Each of these large subfamilies was further divided into three small subfamilies. The three small subfamilies in Yip1p subfamily are represented by YIPF5 (Yip1A), YIPF4, and YIPF6, while those in the Yif1p subfamily are represented by YIF1A, YIPF3, and YIPF1 (Figure 3 and Table 3). For these six smaller subfamilies, Yip1p, Yip4p, Yif1p, and Yip5p were grouped with YIPF5 (Yip1A), YIPF6, YIF1A, and YIPF1, respectively (Supplementary Figure S2 and Table 3). Orthologs for Yip1p, Yif1p, Yip4p, and Yip5p were found in all eukaryotes (Table 2 and Supplementary Table S2) except in diplomonads (e.g., *Giardia intestinalis*) and foraminiferans (e.g., *Reticulomyxa filosa*), in which only a part of orthologs were found at present (Supplementary Figure S2; indicated in white characters). This result strongly suggests that Yip1p, Yif1p, Yip4p, and Yip5p play a fundamental function(s) that is conserved in most eukaryotes. Interestingly, orthologs of YIPF3 and YIPF4 were only found in holozoa, which includes animals (Table 2 and Supplementary Figure S2), but not in holomycota, which includes *S. cerevisiae* and other fungi (Supplementary Table S2 and Supplementary Figure S2) although both holozoa and holomycota are grouped in uniconta. Orthologs of YIPF3 and YIPF4 were found in filasterea and choanoflagellatea, which are single cell organisms closely related to metazoa (Table 2 and Supplementary Figure S2), suggesting that YIPF3 and YIPF4 were evolved in a common ancestor of holozoans that later evolve into metazoa. Curiously, in Ecdysozoa, orthologs of YIPF3 and YIPF4 were found in nematoda, e.g., *Caenorhabditis elegans*, but not in arthropoda, including many insects, i.e., *Drosophila melanogaster* (Table 2 and Supplementary Figure S2). Therefore, it is tempting to speculate that the emergence of YIPF4 and YIPF3, probably by gene duplication from Yip1p and Yif1p or Yip4p and Yip5p, respectively, once played a role in the evolution of metazoans, but those proteins were later lost during the evolution of arthropods.

**TABLE 3:** Summary of nomenclature of YIPF members.

**Systematic name**	**HGNC name**	**Synonyms**	**Localization**	***S. cerevisiae***	**References**
YIPFα1A	YIPF5	Yip1A, FinGER5, SMAP-5	ERGIC	Yip1p	Tang et al., 2001; Yoshida et al., 2008
YIPFα1B	YIPF7	Yip1B, FinGER9	ERGIC		Barone et al., 2015
YIPFα2	YIPF4	FinGER4	*cis*-Golgi	–	Tanimoto et al., 2011
YIPFα3	YIPF6	FinGER6	*Medial-*/*trans*-Golgi/TGN	Yip4p	Soonthornsit et al., 2017
YIPFβ1A	YIF1A	FinGER7, Yif1	ERGIC	Yif1p	Yoshida et al., 2008
YIPFβ1B	YIF1B	FinGER8	ERGIC		Alterio et al., 2015
YIPFβ2	YIPF3	FinGER3, KLIP1	*cis*-Golgi	–	Tanimoto et al., 2011
YIPFβ3A	YIPF1	FinGER1	*Medial-*/*trans*-Golgi/TGN	Yip5p	Soonthornsit et al., 2017
YIPFβ3B	YIPF2	FinGER2	*Medial-*/*trans*-Golgi/TGN		Soonthornsit et al., 2017

In most of the mammals and fishes belonging to Teleostomi, e.g., zebrafish (*Danio rerio*), two close paralogs were found for Yip1p (YIPF5/Yip1A and YIPF7/Yip1B), Yif1p (YIF1A and YIF1B), and Yip5p (YIPF1 and YIPF2), suggesting that gene duplication and subsequent functional divergence occurred to fulfill the needs of vertebrates (Table 2 and Supplementary Figure S2). Curiously, some of these paralogs have not been found in amphibia or aves (birds) at present (Table 2 and Supplementary Figure S2). This may indicate the loss of those paralogs in those organisms, although genome/transcriptome analyses of these organisms may still remain incomplete.

## Proposal of a New Systematic Nomenclature of YIPF Proteins

Human Genome Organization adopted the nomenclature of human YIPF family members based on the original gene names from *S. cerevisiae*, *YIP1* and *YIF1*. HUGO also partly adopted our YIPF family numbering system that is based on the order of the cloning of corresponding cDNAs in our laboratory, except YIF1A and YIF1B (Shakoori et al., 2003). This nomenclature is now out of date and confusing, as it does not reflect the localization or complex forming behavior of the YIPF proteins. In addition, *S. cerevisiae* has a similarly named protein, Yip3p (the mammalian homolog is PRA1), which is also an integral membrane protein with an N-terminal cytoplasmic domain, albeit with only two transmembrane segments (Calero and Collins, 2002; Sivars et al., 2003).

Therefore, we propose renaming the YIPF proteins to clarify the relationship of family members with consideration for their distinct complex formation and localization (Figure 3 and Table 3). (1) The Yip1p homolog is named a subunit (YIPFα) and the Yif1p homolog is named b subunit (YIPFβ), because a pair from each homolog form a complex. (2) Three distinct complexes are formed from special pairs of YIPFα and YIPFβ, and those complexes localize at distinct compartments. Therefore, they are numbered according to their primary localization. The early Golgi (ERGIC) residents are Complex 1 (YIPFα1 and YIPFβ1), the middle Golgi (*cis*-Golgi) residents are Complex 2 (YIPFα2 and YIPFβ2), and the late Golgi (*medial*-/*trans*-Golgi/TGN) residents are Complex 3 (YIPFα3 and YIPFβ3). (3) Two closer paralogs found in vertebrates are differentiated by adding A and B, e.g., YIPFβ3A and YIPFβ3B, because these proteins share a partner YIPF protein and show similar localization, suggesting that they are more similar in their function(s) than other family members (Soonthornsit et al., 2017).

## Conservation of the Primary Structure of YIPF Proteins

When focusing on eukaryotic YIPF proteins, three conserved motifs are found in and around the transmembrane region (Figure 2 and Supplementary Figure S1). They are at (1) the N-terminal cytoplasmic region near the first predicted transmembrane segment [K/R-ϕ/T-x-x-ϕ-ϕ-x-P] (alphabet: single character code of amino acid, f: hydrophobic amino acid, x: any amino acids, alphabets connected with “/” indicated selection of amino acids at that position), (2) the N-terminal side of the first predicted transmembrane segment [D-L/F-x-G/I-P], and (3) the center of the third predicted transmembrane segment [ϕ-ϕ-G-Y-x-ϕ-ϕ-P/G/ϕ-ϕ-ϕ-P/ϕ] (Shakoori et al., 2003). The importance of these conserved motifs is supported by mutation analyses from *S. cerevisiae*. (1) The proline in the second motif and a partially conserved glycine residue found downstream were simultaneously mutated to leucine (P114L, G129E) in the temperature sensitive mutant *yip1-1* (Yang et al., 1998). (2) The glycine at the third position of the third motif was mutated to glutamic acid (G175E) in the temperature sensitive mutant *yip1-2* (Yang et al., 1998). (3) The proline at the eighth position of the third motif was mutated to glycine (P180G) in the lethal mutant *yip1-19* (Chen et al., 2004). These results strongly suggest that the conserved residues in these motifs are functionally relevant. Importantly, all the three conserved motifs consist of proline residue(s). Proline is found in transmembrane segments of many transport proteins and receptors (Brandl and Deber, 1986; Williams and Deber, 1991; Wess et al., 1993). In striking contrast to its structural breaking nature in hydrophilic environment, in a hydrophobic environment mimicking membrane lipid, proline did not largely affect the helical structure of a model peptide (Li et al., 1996; Jacob et al., 1999), on the contrary, it stabilized the helical structure of the peptide in a higher temperature (Li et al., 1996). It was proposed that proline in transmembrane segments function in conformational change of transporters and/or in cationic ligand binding to the transmembrane segments (Brandl and Deber, 1986; Williams and Deber, 1991). Therefore, a primary interest for the future research is the evaluation of the significance of these prolines.

In addition to the motifs conserved in all YIPF members, there are several regions or motifs that are conserved only in a subset of YIPF members (indicated by purple blankets in Supplementary Figure S2). Among these is the [E-P-P-L-E-E] motif, which is conserved in the YIPFα1 (Yip1p) subfamily (indicated by a green blanket). The glutamic acid at the first position of this motif was mutated to lysine (E70K) in the temperature sensitive mutant *yip1-4* (Calero et al., 2003). Upon temperature shift, secretion and growth were blocked concurrent with a massive proliferation of ER membrane, indicating the importance of this motif for the function of the YIPFα1 (Yip1p) subfamily (Heidtman et al., 2003). Similarly, mutation of the glutamic acid at the fifth position of the motif caused lethality in *yip1-6* (E76K) (Chen et al., 2004). In support of this finding, mutation of the corresponding glutamic acid to lysine in human YIPFα1 (Yip1A) (E95K) showed a functional defect in suppressing the formation of multilamellar clustered ER membranes or ER whorls that were caused by the depletion of YIPFα1 (Yip1A) in HeLa cells, which will be discussed again later (Dykstra et al., 2013). Curiously, mutation of the glutamic acid at the first position of this motif to glycine (E89G) did not show a similar functional defect. It is possible that acidic to basic amino acid mutation may be necessary for inducing the observed phenotype, and this possibility and the significance of other motifs should be addressed in future research.

## Complex Formation of YIPF Proteins

As stated above, Yip1p was reported to form a complex with Yif1p for proper function (Matern et al., 2000; Barrowman et al., 2003). Similarly, Yip4p and Yip5p form a complex (Calero et al., 2002). In addition, analysis by us and others revealed that YIPFα1 (Yip1p) sub-ϕamily proteins form a complex with partner YIPFβ1 (Yif1p) sub-ϕamily proteins in human cells, namely, YIPF5 (Yip1A) with YIF1A (Yif1) (Jin et al., 2005; Yoshida et al., 2008), YIPF4 with YIPF3 (Tanimoto et al., 2011), and YIPF6 with YIPF1 or YIPF2 (Soonthornsit et al., 2017). Therefore, it is predicted that, as a general rule, YIPFα1 and YIPFβ1 form a paired complex in order to function (Figure 3 and Table 3).

Analysis in human cells revealed that there are at least three distinct complexes of YIPF proteins; Complex 1 (YIPFα1A–YIPFβ1A) (Jin et al., 2005; Yoshida et al., 2008), Complex 2 (YIPFα2–YIPFβ2) (Tanimoto et al., 2011), and Complex 3 (YIPFα3–YIPFβ3A and YIPFα3–YIPFβ3B) (Soonthornsit et al., 2017). The localization and dynamics of these three complexes are significantly different (Figure 3). Complex 1 localized mainly in the early compartment of the Golgi apparatus (ERGIC) and recycled between the ER and the *cis*-Golgi (Yoshida et al., 2008). Complex 2 localized mainly in the middle compartment of the Golgi apparatus (*cis*-Golgi) and appeared to recycle between the *cis*- and *trans*-Golgi (Tanimoto et al., 2011). Complex 3 localized in the late compartment of the Golgi apparatus (*medial*-/*trans*-Golgi and TGN) and may travel to endosomes (Soonthornsit et al., 2017). These results suggest that YIPFα1–YIPFβ1, YIPFα2–YIPFβ2, and YIPFα3–YIPFβ3 form distinct complexes to carry out their functions. Consistent with this hypothesis, neither YIPFα2 (YIPF4) nor YIPFα1A (YIPF5) co-immunoprecipitated with YIPFα3 (YIPF6) (Soonthornsit et al., 2017).

Interestingly, si-RNA knockdown of a YIPFα protein specifically reduced the presence of their special partner YIPFβ protein(s) in the same complex, strongly suggesting an exclusive relationship between these family members. Namely, the knockdown of YIPFα1A (YIPF5) reduced YIPFβ1A (YIF1A) (Yoshida et al., 2008), the knockdown of YIPFα2 (YIPF4) reduced YIPFβ2 (YIPF3) (Tanimoto et al., 2011), and the knockdown of YIPFα3 (YIPF6) reduced YIPFβ3A (YIPF1) and YIPFβ3B (YIPF2) (Soonthornsit et al., 2017). Our results showed that knockdown of YIPFα1, YIPFα2, and YIPFα3 only affected YIPFβ1, YIPFβ2, and YIPFβ3, respectively, but not the other family members (Soonthornsit et al., 2017). These results suggest that the expression of a YIPFα protein is specifically and exclusively regulated by the expression of a partner YIPFβ protein. Taken together, this strongly suggests that a YIPFα protein has a specific partner YIPFβ, and these two molecules form a complex that serves as the basic unit for their function. This is supported by the finding that two pairs of YIPF proteins (or more precisely, transcripts or genes coding those proteins) (YIPFα1 and YIPFβ1 or YIPFα3 and YIPFβ3) are found in virtually all eukaryotes (Table 2 and Supplementary Table S2). Exceptionally, two close homologs were shown to share a partner protein, i.e., YIPFβ3A (YIPF1) and YIPFβ3B (YIPF2) with YIPFα3 (YIPF6) (Soonthornsit et al., 2017). This result suggests that YIPFα1A and YIPFα1B or YIPFβ1A and YIPFβ1B may also share partner proteins, but this possibility must be confirmed in future research. In addition, whether *S. cerevisiae* Yip1p and Yif1p and Yip4p and Yip5p are localized to the early and late Golgi compartments, respectively, must also be confirmed.

Yip1 domain family proteins were observed in higher order oligomers in mild detergent extract of HeLa cells. YIPFα1A–YIPFβ1A were estimated to form ∼4–8 mer complexes (Yoshida et al., 2008) while YIPFα2–YIPFβ2 form ∼4–16 mer complexes (Tanimoto et al., 2011). These results suggest that a tetramer consisting of YIPFα and YIPFβ, which is most probably formed from two of each molecule, is the minimum functional unit of the YIPF complex. It is possible that a tetramer sub-complex mainly associates homogeneously to form higher order oligomers, although oligomers consisting of mixed sub-complexes may also exist.

## Function of YIPF Proteins in Animal Cells

Soon after the identification of *S. cerevisiae* Yip1p, human YIPFα1A (Yip1A) was cloned (Tang et al., 2001; Kano et al., 2004). The protein was shown to localize at the ER exit site and its cytoplasmic domain to interact with COPII components Sec23 and Sec24. YIPFα1A was efficiently incorporated into COPII vesicles and the overexpression of the cytoplasmic domain of YIPFα1A induced the disruption of the Golgi apparatus and inhibited the transport of VSV-G, a transmembrane marker protein, to the Golgi apparatus (Tang et al., 2001). These results suggest that YIPFα1A functions at ER exit sites and is involved in the recruitment of selected soluble and membrane cargo proteins to COPII vesicles. Under mitotic conditions, YIPFα1A was delocalized from the ER exit sites and spread diffusely throughout the ER in parallel with the dissociation of COPII components from the ER exit sites (Kano et al., 2004). This result was consistent with the close relationship of YIPFα1A to COPII components.

Later, we found that YIPFα1A (YIPF5) formed a complex with YIPFβ1A (YIF1A) and mainly localized at the early Golgi compartment (ERGIC and some in *cis*-Golgi) (Yoshida et al., 2008). We also found that YIPFα1A and YIPFβ1A were recycled between the ER and the Golgi apparatus. Our observation is consistent with former reports, because a part of YIPFα1A had to exist at the ER exit site during its trafficking between the ER and the Golgi apparatus. Knockdown of YIPFα1A or YIPFβ1A induced significant fragmentation of the Golgi apparatus with an accumulation of vesicles around the shortened stacked cisternae without significantly affecting anterograde transport (Yoshida et al., 2008; Kano et al., 2009). These results argue against the role of YIPFα1A in COPII-dependent ER to Golgi transport. In contrast, Lee’s group reported that the depletion of YIPFα1A by si-RNA reduced ER to Golgi transport and induced an abnormal multi-lamellar ER structure, supporting the original idea that YIPFα1A functions during COPII vesicle budding from the ER (Dykstra et al., 2010).

The key to understanding this discrepancy may be the expression levels of YIPFα1B/Yip1B and/or YIPFβ1B/Yif1B. It is probable that YIPFα1B and/or YIPFβ1B compensated for the function of YIPFα1A and/or YIPFβ1A because YIPFα1B and YIPFβ1B are the closest homologs of YIPFα1A and YIPFβ1A, respectively. Because the expression levels of YIPFα1B and YIPFβ1B were not determined in any of the above discussed analyses, it is possible that higher expression of YIPFα1B or YIPFβ1B compensated for the loss of YIPFα1A or YIPFβ1A in our hands. However, this possibility does not necessarily mean YIPFα1A and YIPFα1B, YIPFβ1A and YIPFβ1B have completely overlapping roles. The functional difference between YIPFα1A and YIPFα1B is of particular interest because YIPFα1B/Yip1B has been reported to be expressed in muscle cells with a parallel loss of YIPFα1A/Yip1A (Barone et al., 2015). Similarly, YIPFβ1B may have a special role in cargo transport in neuronal cells (Carrel et al., 2008; Alterio et al., 2015).

Aside from these problems, there was an interesting report showing that the knockdown of YIPFα1A/Yip1A induced the dissociation of Rab6 from the Golgi membrane and reduced COPI independent Golgi to ER retrograde transport (Kano et al., 2009). However, there are difficulties in interpreting the results; firstly, because there are three isoforms of Rab6, Rab6A, Rab6A′, and Rab6B, it is unknown which of them were affected in the study. Secondly, the localization of Rab6, which was likely at the *trans*-side of the Golgi apparatus (Antony et al., 1992), was clearly different from YIPFα1A, which was at the ERGIC (Yoshida et al., 2008). Therefore, the mechanism by which knockdown of YIPFα1A affected the localization of the isoform(s) of Rab6 must be clarified to evaluate the significance of this study. Nevertheless, the finding gives us a clue toward clarifying the molecular mechanism and function of the Rab6 dependent, COPI independent retrograde transport pathway which remains poorly understood (Liu and Storrie, 2012).

*Brucella* is a pathogen that invades and replicates inside cells (Taguchi et al., 2015). It has been shown that *Brucella* induced the formation of, and resided in, membrane-bound structures called *Brucella*-containing vacuole (BCV) in the cytoplasm, which eventually fused with ER exit sites to form the replication machinery. It was shown that the IRE1-dependent unfolded protein response was necessary for the induction of BCV. Intriguingly, YIPFα1A/Yip1A was shown to be required for the activation of IRE1 following BCV formation and *Brucella* replication. In addition, knockdown of YIPFα1A inhibited the oligomer formation and activation of IRE1 induced by tunicamycin, which is a general stress inducer. Therefore, it is hypothesized that YIPFα1A is involved in stress-induced IRE1 activation in the ER, which is also induced by *Brucella* infection. A subsequent study revealed that YIPFα1A was also involved in the activation of stress adaptations and survival in cancer cells (Taguchi et al., 2017). Knockdown of YIPFα1A reduced the activation of IRE1 and also PERK, which are upstream regulators of the ER stress response and promote cell survival. It is possible that YIPFα1A functions as a chaperone for transmembrane proteins and promotes oligomerization and activation of IRE1 and PERK. This possibility must be evaluated in future studies.

Interestingly, YIPFα2/YIPF4 was shown to interact with E5 proteins from several different types of human papillomaviruses, including HPV-16 and HPV-18 (Müller et al., 2015). Papillomavirus E5 protein is a small multi-span transmembrane protein that has oncogenic activities in epidermal cells (Venuti et al., 2011). Among human papillomaviruses, types 16 (HPV-16) and 18 (HPV-18) have attracted interest because of their high risk for inducing cancer after infection. E5 of HPV-16 was shown to localize at the ER and Golgi apparatus under lower level expression, and also at the plasma membrane under high level expression. YIPFα2 and E5 of HPV-16 were shown to interact at the transmembrane region (Müller et al., 2015). However, these results must be interpreted with caution, because two opposing membrane topologies have been proposed for E5: one predicts the N-terminus to be exposed to the cytosol with the C-terminus exposed to the lumen of the Golgi apparatus (Hu and Ceresa, 2009), while the other predicts the N-terminus to be exposed to the lumen of the Golgi apparatus with the C-terminus exposed to the cytosol (Krawczyk et al., 2010). The tagging of proteins in the above study may not reproduce the native topology of the E5 protein. Intriguingly, YIPFα2 was found to decrease in calcium differentiated human foreskin keratinocytes, while the presence of viral genome in the cells rescued the expression of YIPFα2 and the presence of E5 was not necessary for this effect. The significance of the change in YIPFα2 expression during keratinocyte differentiation and its relationship with HPV infection should be a subject for future investigation.

A mutation in YIPFα3/YIPF6 that caused truncation of the coding sequence, deleting the entire transmembrane region, was shown to render mice susceptible to colitis when the mice were fed a non-toxic dose of dextran sodium sulfate, a model for inflammatory bowel disease (Brandl et al., 2012). Pathological and biochemical analyses revealed that the number of Paneth cells and goblet cells was reduced in the mutant mice. In addition, the secretory granules were smaller and more disorganized in the Paneth cells. Similarly, mucin granules were smaller and more irregular in size in the goblet cells. In accordance, the mucin content of the colon was reduced. These results suggest that YIPFα3 is involved in the synthesis and/or exocytotic transport of mucin in goblet cells in the mutant mice. It is possible that the loss of YIPFα3 damaged either export of mucin from the *trans*-Golgi or maturation of mucin granules at TGN in the goblet cells. A similar defect in the secretory pathway is hypothesized to occur in Paneth cells. Importantly, and rather surprisingly, the mutant mice were viable and grew more or less normally under normal feeding conditions up to 7 days after the birth, suggesting the loss of YIPFα3 did not cause severe developmental or functional defects (Brandl et al., 2012). This result suggests that the function of YIPFα3 is cell type specific, or the effect of the loss of YIPFα3 was somehow compensated for in most other cell types. In other words, it is likely that differentiated goblet cells and Paneth cells, but not other cells, are highly dependent on the function of YIPFα3. Consistently, the depletion of YIPFα3 by si-RNA did not induce a significant effect either on the ER and Golgi structures or on ER to plasma membrane transport in HeLa cells or HT-29 cells that secrete mucin (Soonthornsit et al., 2017). We hope for a more detailed analysis of the YIPFα3 mutant mice to be carried out in order to obtain further clues about the function of YIPFα3.

It was recently reported that the expression of YIPFα3 was increased in prostate cancer cells that showed bone metastasis and became resistant to castration (Djusberg et al., 2017). In these cells, the androgen receptor gene was amplified together with the YIPFα3 gene, which is located near the androgen receptor gene on the X chromosome (Xq12). The increase in these expression levels was thought to contribute to the malignant phenotype of the cancer cells (Vainio et al., 2012; Djusberg et al., 2017). Curiously, over-expression of YIPFα3 in 22Rv1 cells, which expresses high androgen receptor activity, reduced, instead of increased, cell proliferation. Therefore, whether and how the increase of YIPFα3 affects the malignancy of cancer cells remain unclear. Intriguingly, an increase in the number of ∼83 nm diameter extracellular vesicles, which may function as exosomes, was also induced by the over expression of YIPFα3. Whether this effect is also found in other cell types and how this increase in presumed exosomes is induced are of special interest for elucidating the function of YIPFα3.

YIPFβ1A/YIF1A was shown to interact with VAPB, a mutant of which (VAPB–P56S) has been linked to motor neuron degeneration in amyotrophic lateral sclerosis type 8 (ALS8) (Kuijpers et al., 2013). VAPB is a type II ER membrane protein, which interacts with lipid exchange and lipid-sensing proteins that have FFAT motifs. VAPB is thus thought to be involved in the organization of lipid metabolism and non-vesicular lipid transfer to and from the ER. The chronic expression of VAPB–P56S induced small inclusions scattered around the cytoplasm where the mutant protein accumulated. These inclusions were shown to be formed from and connected to the ER (Fasana et al., 2010). YIPFβ1A was shown to bind to both the wild type and the mutant form of VAPB (VAPB–P56S). Interestingly, YIPFβ1A and YIPFβ1B/YIF1B accumulated in the inclusions induced by VAPB–P56S. Furthermore, overexpression of VAPB induced a dispersal of YIPFβ1A throughout the neuron while the knockdown of VAPB induced an accumulation of YIPFβ1A around the Golgi area. These results suggest that the interactions of VAPB with YIPFβ1A and possibly also with YIPFβ1B have a significant role in the pathology of VAPB–P56S (Kuijpers et al., 2013).

YIPFβ1B/YIF1B was found to interact with 5-HT_1A_R, one of the serotonin receptors localized at the plasma membrane of soma and dendrites of neurons in the central nervous system (Carrel et al., 2008). 5-HT_1A_R is a G protein coupled receptor and a major target of anti-depressant drugs, and how it is delivered to a specialized region of the neuron has attracted a medical interest (Barnes and Sharp, 1999). 5-HT_1A_R showed yeast two-hybrid interaction with YIPFβ1B and the depletion of YIPFβ1B in primary neurons specifically prevented the delivery of 5-HT_1A_R to distal portions of dendrites (Carrel et al., 2008). YIPFβ1B was hypothesized to support the delivery of 5-HT_1A_R specifically because the loss of YIPFβ1B did not affect the delivery of other receptors, such as sst2AR, P2X2R, and 5-HT_3A_R. YIFPβ1B was shown to localize mainly in the ERGIC, similar to YIPFβ1A. Yoshida et al. (2008) and Alterio et al. (2015) raised the question of how an ERGIC protein determines the delivery of cargo proteins to specific regions of the plasma membrane. One possibility is that the loss of YIFPb1B perturbed the proper processing of 5-HT_1A_R at the Golgi apparatus, including glycosylation, leading to mis-sorting of the 5-HT_1A_R at the *trans*-Golgi/TGN. It is possible that YIPFβ1B is directly involved in the processing of 5-HT_1A_R by delivering 5-HT_1A_R to processing enzymes. Alternatively, YIPFβ1B may be indirectly involved in the processing of 5-HT_1A_R, supporting the proper localization of processing enzymes in the Golgi apparatus. It is also possible that YIFPβ1B functions as a molecular chaperone helping 5-HT_1A_R to form a proper conformation that is necessary for its correct delivery, or helping 5-HT_1A_R assemble with other factors that support the proper delivery of target molecules.

It was recently reported that YIPFβ1/Yif1 and YIPFα1/Yip1 have an essential role in dendrite pruning in *Drosophila* (Wang et al., 2018). As in mammalian cells, *Drosophila* YIPFα1 and YIPFβ1 form a complex and localize mainly in the ERGIC and the Golgi apparatus. Interestingly, the Golgi apparatus was fragmented in ddaC sensory neurons with truncated mutants of YIPFα1 (Yip1) or YIPFβ1 (Yif1), while these proteins were dispensable for viability or apoptosis. How YIPFα1 and YIPFβ1 induce dendrite pruning in *Drosophila* remains unclear.

## Mechanisms of YIPF Protein Function and Future Directions for Research

After two decades of studies following discovery of the first YIPF proteins (Yip1p) in *S. cerevisiae*, the molecular mechanisms of YIPF protein function still remain obscure. It has been difficult to analyze the family in mammalian cells, most likely because of the overlapping functions of YIPF family members. *S. cerevisiae* only has four family members and is thought to be better suited for the analysis for YIPF proteins, although a decade without any reports on the function of yeast YIPF proteins implies that things have not been so easy, even in *S. cerevisiae*. In this aspect, the analysis of YIPF proteins in any other available model organisms will face difficulty resulting from the expected overlapping functions, because virtually all eukaryotes have at least four family members. Therefore, even *D. melanogaster* may not be an ideal model organism because it also has four family members.

Four family members and two distinct complexes were identified in *S. cerevisiae*; Complex 1; YIPFα1 (Yip1p) and YIPFβ1 (Yif1p) and Complex 3; YIPFα3 (Yip4p) and YIPFβ3 (Yip5p). From the analysis of mammalian family members, Complex 1 is predicted to function in ER to Golgi transport (YIPFα1, YIPFβ1) and Complex 3 in transport between the Golgi and downstream compartments such as endosomes (YIPFα3, YIPFβ3). This idea is supported by the finding that abnormal vacuolar morphology was observed with the null mutants of *YIP4* and *YIP5* (Michaillat and Mayer, 2013), and endocytosis decreased in the null mutant of *YIP4* (Burston et al., 2009). A more detailed analysis of these null mutants will provide more clues toward understanding the function of these complexes.

An earlier study suggested that YIPF proteins were candidates for classical yeast two hybrid analysis when tagged on their N-termini (Yang et al., 1998; Ito et al., 2000; Matern et al., 2000; Uetz et al., 2000; Calero et al., 2001, 2002; Calero and Collins, 2002; Shakoori et al., 2003). However, several trials in our laboratory at library screening using yeast two hybrid analysis to identify binding partners of human YIPF proteins produced no likely candidates. In particular, neither Rab1 nor Rab11, which are orthologs of Ypt1p and Ypt31p that interact with yeast Yip1p, Yif1p, Yip4p, and Yip5p, was picked up either by classical yeast two hybrid analysis or by pull down analysis. Therefore, no direct evidence that YIPF proteins function with Rab/Ypt GTPases has been obtained in mammalian cells so far. It was proposed that Yip1p binds the GDP bound form of Ypt1p or Ypt31p because Yip1p did not show yeast two hybrid interaction with GTPase deficient mutants of Ypt1p or Ypt31p (Yang et al., 1998). To test this possibility in mammalian cells, we tried to detect the interaction of human YIPF proteins with a human or rat GDP binding mutant of Rab1 by yeast two-hybrid analysis, but this has again been unsuccessful to date. It is possible that design of bait and/or pray constructs was not adequate to allow the access of mammalian Rab proteins and YIPF proteins in our system. Therefore, redesigning of the bait and/or pray constructs or use of other analytical system is necessary to evaluate the interaction of mammalian Rab proteins and YIPF proteins. However, we cannot exclude the possibility that YIPF proteins evolved to dispense with the interaction to Rab proteins for their functions in mammalian cells.

The only significant interactions of human proteins found by classical yeast two hybrid analysis were between ArfGAP1 and YIPFβ1A (YIF1A/FinGER7) or YIPFβ1B (YIF1B/FinGER8) (Akhter et al., 2007). Interestingly, this interaction was mediated by the first ALPS motif of ArfGAP1, which was hypothesized to sense curvature of the lipid bilayer (Bigay et al., 2005). However, experiments in our laboratory using detergent extract of HeLa cells have not been able to confirm these interactions. It has been proposed that the ALPS motif binds highly curved small liposome membranes by inserting hydrophobic bulky residues into a loosely packed lipid layer. The ALPS motif was unstructured in its membrane unbound soluble state, but formed an amphipathic helix in the membrane bound state (Bigay et al., 2005). Therefore, it is possible that the conformation of ArfGAP1 could not be reproduced in the presence of detergent, disrupting the interaction of ArfGAP1 and YIPFβ1A or YIPFβ1B. Future work using reconstituted liposomes may overcome this limitation.

As we discussed above, YIPF proteins form higher order oligomers consisting of at least four YIPF protein molecules. Because one YIPF protein has five transmembrane segments, a tetramer is predicted to have 20 transmembrane segments. There are many hydrophilic amino acid residues in these transmembrane segments suggesting that YIPF proteins function as channels, transporters, or transmembrane receptors. If so, the existence of distant homologs in prokaryotic cells, in which no membrane trafficking pathway has been developed, may be informative. It is possible that these prokaryotic homologs function in the transport of some hydrophilic solute (s), such as ions and organic molecules. Alternatively, they may function as membrane receptors responding to extracellular molecules. It is likely that YIPF proteins have a common function with their prokaryotic homologs in this respect, aside from their function in membrane trafficking pathways. It is even possible that their function as a transporter, a channel, or a receptor is their main function in eukaryotes and the observed interactions between YIPF proteins and Ypt/Rab GTPases and other molecules involved in membrane trafficking serve to control the proper localization of these proteins.

Indeed, transmembrane segments of YIPF proteins consist of conserved proline residues (Figure 2), which is found or even conserved in many transporters (Brandl and Deber, 1986; Wess et al., 1993). Furthermore, bioinformatics analysis using SCOOP showed that the Yip1 domain has significant similarity to known transporters (Bateman and Finn, 2007). The Yip1 domain is now grouped as clan Yip1 (CL0112), which contains the following seven family members in the Pfam database: DUF1048, DUF1129, DUF1189, DUF1282, DUF1700, YIF1, and Yip1 (El-Gebali et al., 2019). Among these, DUF1282 (pfam06930), which includes bacterial Yip1p domain families, has similarity with the sulfate permease family (PF00916). On the other hand, DUF1129 (pfam06570) has similarity to Sugar_tr (PF00083), which includes transporters responsible for moving various carbohydrates, organic alcohols, and acids in a wide range of prokaryotic and eukaryotic organisms. DUF1700 has similarity to VIT1 (PF01988), which includes a group of putative vacuolar ion transporters. These results support the possibility that YIPF protein plays a role as a transporter.

The Golgi apparatus is a factory where glycosylation, sulfation, and phosphorylation of secretory and membrane proteins occur. Substrates and by-products have to be transported into and out of the lumen of the Golgi apparatus to sustain many reactions. Many transporters that support these function have already been identified (Berninsone and Hirschberg, 2000; Hirschberg, 2013). However, many remain undiscovered, including those responsible for the transport of inorganic phosphate, which is produced from nucleotide di-phosphate to reinforce the one-way reaction of sugar transfer to substrates. Physiologically identified transporters GOLAC-1 and GOLAC-2 are possible candidates to fulfill this function (Nordeen et al., 2000; Thompson et al., 2006). An anion channel that functions for the acidification of the Golgi lumen (GPHR) was proposed to be GOLAC-2 (Maeda et al., 2008). On the other hand, a molecule responsible for the function of GOLAC-1 has not yet been identified. Is it possible that YIPF proteins function as GOLAC-1?

One other possibility is that YIPF proteins function as membrane protein chaperones, assisting with the conformational maturation and/or complex formation of transmembrane proteins. The results that YIPFα1A was involved in the oligomerization and activation of IRE1 support this possibility (Taguchi et al., 2015, 2017). Finally, there is a possibility that YIPF proteins function to specify the identity of domains of the Golgi apparatus. Namely, the presence of YIPF Complex 1 (YIPFα1–YIPFβ1), Complex 2 (YIPFα2–YIPFβ2), and Complex 3 (YIPFα3–YIPFβ3) determine the identity of early (ERGIC), middle (*cis*-Golgi), and late Golgi (*medial*-*/trans*-Golgi/TGN) compartments, respectively (Figure 3). Golgi resident transmembrane proteins may be anchored to YIPF protein complexes through interactions at transmembrane regions. The dynamics of YIPF proteins support this possibility. After Brefeldin A treatment, most Golgi resident proteins are transported back to the ER, while TGN proteins are transported to the endosomal compartment (Lippincott-Schwartz et al., 1991). YIPF proteins, however, did not travel with these Golgi proteins but remained in distinct cytoplasmic vesicular structures after treatment with Brefeldin A (Yoshida et al., 2008; Tanimoto et al., 2011; Soonthornsit et al., 2017). Therefore, YIPF proteins have the capacity to resist vesicular flow, and this capacity is well-suited for determining compartment identities. To evaluate this possibility, the mechanisms which determine the localization of the three YIPF subcomplexes must be better understood.

## Author Contributions

All authors listed have made a substantial, direct and intellectual contribution to the work, and approved it for publication.

## Conflict of Interest Statement

The authors declare that the research was conducted in the absence of any commercial or financial relationships that could be construed as a potential conflict of interest.

## Footnotes

1 https://www.yeastgenome.org/

## Supplementary Material

FIGURE S1 Multiple sequence alignments produced by CLUSTAL W are shown. The five predicted transmembrane segments are indicated on the top (TM1–TM5). Orthologs of family members are grouped and indicated by colored background on the left. YIPF proteins are grouped in two subfamilies (a and b) that are further sub-grouped into three (1–3). In higher chordata, YIPFα1, YIPFβ1, and YIPFβ3 were further sub-divided into A and B (refer text and Table 3 for the definition). The conserved motifs are indicated by red (conserved in all YIPF members) or green (conserved in YIPFα1/Yip1p) lines on the bottom and blankets in the same color on the aligned sequences. Other conserved regions with unknown significance are indicated by purple blankets. The YIPF protein sequences were identified by BLAST search using human and *S. cerevisiae* YIPF protein sequences. A representative species for each phylum or class was selected for the analysis to simplify the results. Duplicated data sets and divergent isoform sequences were omitted.

FIGURE S2 Multiple sequence alignments were produced by CLUSTAL W, as described in Supplementary Figure S1 and a phylogenetic tree with boot strap values was drawn by NJplot version 2.3. Orthologs of family members are grouped and indicated by colored background as in Supplementary Figure S1.

TABLE S1 Genetic interactors of budding yeast YIPF proteins.

TABLE S2 Eukaryotic species in which all the ortholog of Yip1p, Yif1p, Yip4p, and Yip5p were found.

TABLE S3 Prokaryote species in which YIPF homologs are found.

## Abbreviations

CDD: Conserved Domain Database
ERGIC: ER–Golgi intermediate compartment
HUGO: Human Genome Organization
SGD: *Saccharomyces* Genome Database
TGN: *trans*-Golgi network
YIPF: Yip1 domain family.
