# Supplementary figures and images for: Characteristics and Functions of the Yip1 Domain Family (YIPF), Multi-Span Transmembrane Proteins Mainly Localized to the Golgi Apparatus
Source: Front Cell Dev Biol. 2019 Jul 30;7:130. doi: 10.3389/fcell.2019.00130 (PMC6682643; doi:10.3389/fcell.2019.00130)

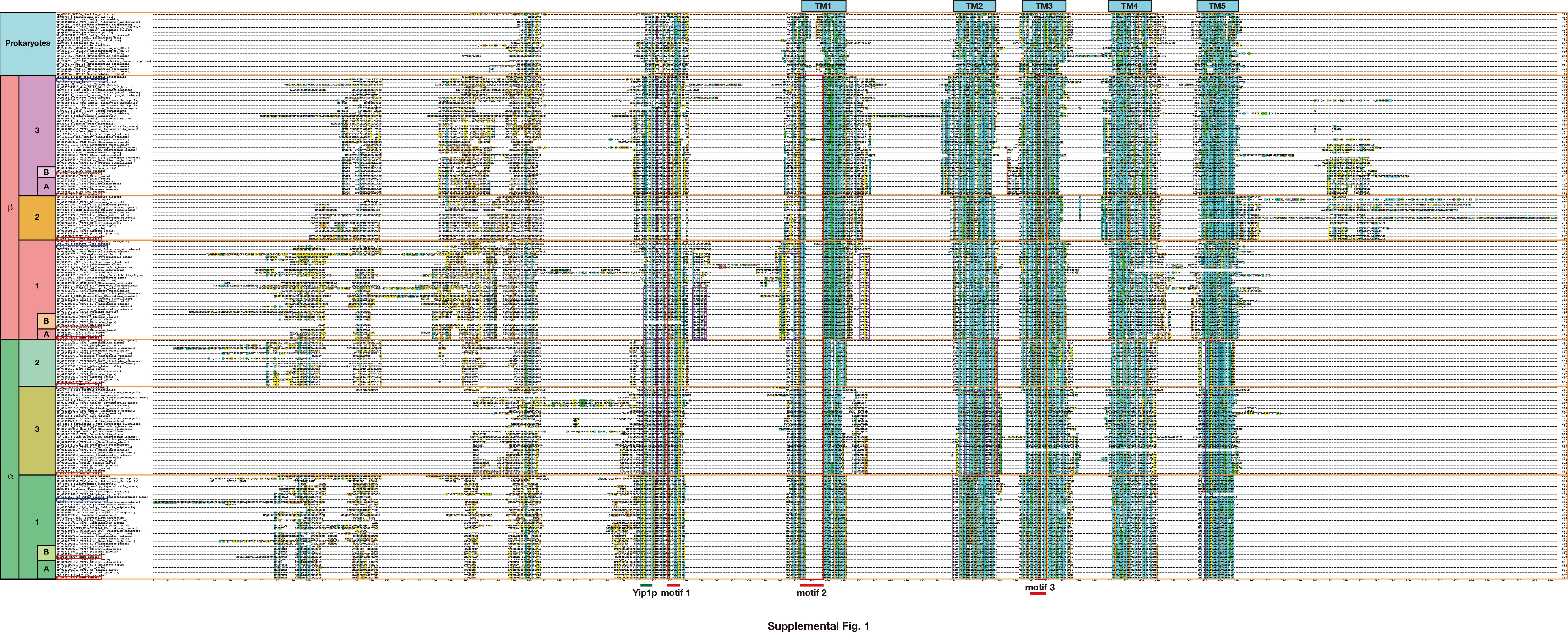

Supplement: FIGURE S1 — Multiple sequence alignments produced by CLUSTAL W are shown. The five predicted transmembrane segments are indicated on the top (TM1–TM5). Orthologs of family members are grouped and indicated by colored background on the left. YIPF proteins are grouped in two subfamilies (a and b) that are further sub-grouped into three (1–3). In higher chordata, YIPFα1, YIPFβ1, and YIPFβ3 were further sub-divided into A and B (refer text and Table 3 for the definition). The conserved motifs are indicated by red (conserved in all YIPF members) or green (conserved in YIPFα1/Yip1p) lines on the bottom and blankets in the same color on the aligned sequences. Other conserved regions with unknown significance are indicated by purple blankets. The YIPF protein sequences were identified by BLAST search using human and S. cerevisiae YIPF protein sequences. A representative species for each phylum or class was selected for the analysis to simplify the results. Duplicated data sets and divergent isoform sequences were omitted. [file Image_1.JPEG]

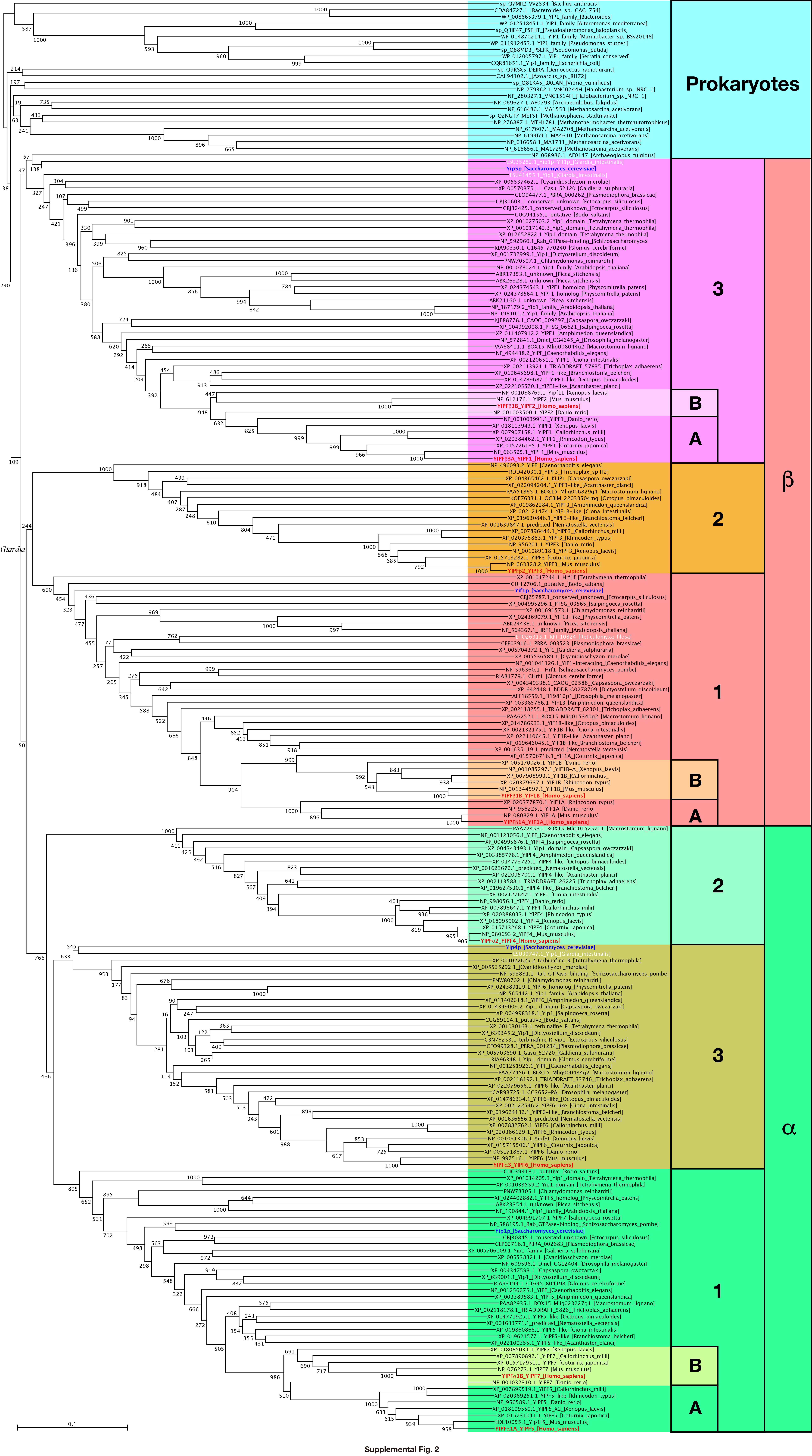

Supplement: FIGURE S2 — Multiple sequence alignments were produced by CLUSTAL W, as described in Supplementary Figure S1 and a phylogenetic tree with boot strap values was drawn by NJplot version 2.3. Orthologs of family members are grouped and indicated by colored background as in Supplementary Figure S1. [file Image_2.JPEG]
