# Supplementary material for: Characteristics and Functions of the Yip1 Domain Family (YIPF), Multi-Span Transmembrane Proteins Mainly Localized to the Golgi Apparatus
Source: Front Cell Dev Biol. 2019 Jul 30;7:130. doi: 10.3389/fcell.2019.00130 (PMC6682643; doi:10.3389/fcell.2019.00130)
Supplement: TABLE S1 — Genetic interactors of budding yeast YIPF proteins. [file Table_1.DOCX]

Supplemental Table 1. Genetic interactors of budding yeast YIPF proteins

Genetic interactors of all YIPF genes were combined and categorized according to functional significance. Only the categories of interests are shown. Physical interactors are indicated in bold letters. Refer SGD for the full dataset and additional information.

| Category | Gene name |
| --- | --- |
| Ypt | ***SEC4****,* ***VPS21****,* ***YPT1****, YPT11,* ***YPT31****, YPT32,* ***YPT52****, YPT6* |
| PX domain | *Vps5* |
| Ypt related | *BET4, BTS1, CCZ1, GDI1, MRS6, RGP1, ROY1, RIC1, SEC2, SRO7* |
| SNARE | *GOS1, SEC20, SEC22, SNC2, TLG1, TLG2, USE1, VTI1* |
| COPI | *COP1, RET2, RET3, SEC21, SEC26, SEC27, SEC28* |
| COPII | *SEC12,* ***SEC13****, SEC16,* ***SEC23****,* ***SEC24****, SEC31, SFB2, SFB3* |
| COPII cargo | *ERV14, GOT1,* ***YOS1****, YIP3* |
| Retromer | *VPS29, VPS35* |
| ARFGAP | ***GCS1****, GLO3* |
| p23 | *EMP24, ERV25* |
| TRAPP complex | *BET3, BET5, TRS20, TRS23, TRS85* |
| COG complex | *COG3, COG5, COG8* |
| GET complex | *GET1, GET2, GET3* |
| ESCRT complex | *SRN2, STP22* |
| GARP complex | *VPS51, VPS53* |
| Golgi localized | *AVL9, BUG1, DRS2, SAC1, SYS1, USO1, VPS74* |
| Membrane traffic | *APS1, ARL, CLC1, INP53, LST8, NEO1, NPR3, SLA2, SWA2, VAM10* |
| Translocon | *SEC62, SEC63, SEC66, SRP102* |
| ER localized | *ARV1, CSG2, EPO1, ERI1, ERO1, HMX1, ICE2, IRE1, KAR2, LHS1, PHO86, RFT1, SEC11, SND2, VPS13,* ***YOP1*** |
| Nuclear pore, envelope, import | *AIM4, APQ12, BRL1, BRR6, CEX1, GSP1, LOS1, NUP116, NUP120, NUP159, NUP170, NUP192, MTR10, RNA1* |
| Endosome, vacuole | *COS7, FAB1, HSE1, MEH1, MON2, RNY1, SNN1, VMA3, VMA8, VMA9, VMA21, VPS1, ZRC1* |
| Autophagy | *ATG2, ATG15,* ***VPS30*** |
| Glycosylation | *CWH41, FKS1, HKR1, HOC1, KRE5, MNN10, MNN11, MNN2, OST3, TED1, WBP1* |
| GPI anchor related | *BST1, CDC1, CWH43, GAA1, GAB1, GAS3, GPI10, GPI12, GPI13, GPI15, GPI17, GPI19, GPI2, GPI8, GUP1, GWT1, LAS21, PER1* |
| Lipid metabolism | *ANY1, CSH1, CHO2, ELO2, LCB2, LDB16, OPI3, ORM2, PHS1, RAM2, RER2, RVS161, SCS7, SEI1, SUR1, TSC3, TSC13, UPS1* |
| Transporter | *ALR2, ITR2, PHO84, PMR1, SPF1* |
