# Supplementary material for: Characteristics and Functions of the Yip1 Domain Family (YIPF), Multi-Span Transmembrane Proteins Mainly Localized to the Golgi Apparatus
Source: Front Cell Dev Biol. 2019 Jul 30;7:130. doi: 10.3389/fcell.2019.00130 (PMC6682643; doi:10.3389/fcell.2019.00130)
Supplement: TABLE S2 — Eukaryotic species in which all the ortholog of Yip1p, Yif1p, Yip4p, and Yip5p were found. [file Table_2.DOCX]

Supplemental Table 2. Eukaryotic species in which all the orthologue of Yip1p, Yif1p, Yip4p and Yip5p were found

| Taxon | (Kingdom) | (Phylam) | (Class) | Species |
| --- | --- | --- | --- | --- |
| Excavata | Euglenozoa |  | Kinetoplastida | *Bodo saltans* |
| SAR | Stramenopiles | Heterokontophyta | Phaeophyceae | *Ectocarpus siliculosus* |
|  | Alveorates | Ciliophora | Oligohymenophorea | *Tetrahymena thermophila* |
|  | Rhizaria | Cercozoa | Phytomyxea | *Plasmodiophora brassicae* |
| Archaeplastida | Rhodophyta | | Bangiophyceae | *Galdieria sulphuraria*  *Cyanidioschyzon_merolae* |
|  | Viridiplantae | Chlorophyta | Chlorophyceae | *Chlamydomonas reinhardtii* |
|  |  | Streptophyta | Bryopsida | *Physcomitrella patens* |
|  |  |  | Acrogymnospermae | *Picea sitchensis* |
|  |  |  | Magnoliophyta | *Arabidopsis thaliana* |
| Uniconta | Amebozoa | Mycetozoa | Dictyostelids | *Dictyostelium discoideum* |
|  | Opisthokonta | Holomycota | Mucoromycota | *Glomus cerebriforme* |
|  |  |  | Dikarya | *Saccharomyces cerevisiae*  *Schizosaccharomyces pombe* |
