# Supplementary material for: Characteristics and Functions of the Yip1 Domain Family (YIPF), Multi-Span Transmembrane Proteins Mainly Localized to the Golgi Apparatus
Source: Front Cell Dev Biol. 2019 Jul 30;7:130. doi: 10.3389/fcell.2019.00130 (PMC6682643; doi:10.3389/fcell.2019.00130)
Supplement: TABLE S3 — Prokaryote species in which YIPF homologs are found. [file Table_3.DOCX]

Supplemental Table 3. Prokaryote species in which YIPF homologues are found

| Superkingdom | phylum | class | Species |
| --- | --- | --- | --- |
| Bacteria | Bacteroidetes | Bacteroidia | *Bacteroides* |
|  | Proteobacteria | Betaproteobacteria | *Azoarcus* |
|  |  | Gammaproteobacteria | *Alteromonas mediterranea*  *Escherichia coli*  *Marinobacter*  *Pseudoalteromonas haloplanktis*  *Pseudomonas stutzeri*  *Pseudomonas putida*  *Serratia*  *Vibrio vulnificus* |
|  | Firmicutes | Bacilli | *Bacillus anthracis* |
|  | Deinococcus-Thermus | Deinococci | *Deinococcus radiodurans* |
|  | Thermotogae | Thermotogae | *Fervidobacterium nodosum* |
| Archaea | Euryarchaeota | Archaeoglobi | *Archaeoglobus fulgidus* |
|  |  | Methanobacteria | *Methanosphaera stadtmanae*  *Methanothermobacter thermautotrophicus* |
|  |  | Methanococci | *Methanocaldococcus jannaschii* |
|  |  | Methanomicrobia | *Methanosarcina acetivorans* |
|  |  | Halobacteria | *Halobacterium* |
